# Determinants of Self-Rated Health in a Representative Sample of a Rural Population: A Cross-Sectional Study in Greece

**DOI:** 10.3390/ijerph9030943

**Published:** 2012-03-16

**Authors:** Christina Darviri, Georgia Fouka, Charalambos Gnardellis, Artemios K. Artemiadis, Xanthi Tigani, Evangelos C. Alexopoulos

**Affiliations:** 1 Postgraduate Course Stress Management and Health Promotion, School of Medicine, University of Athens, Soranou Ephessiou Street, 4, 11527 Athens, Greece; Email: cdarviri@med.uoa.gr (C.D.); artemiad@med.uoa.gr (A.K.A.); ecalexop@med.uoa.gr (E.C.A.); 2 Technological Education Institute of Athens, Ag Spyridonos Street, Egaleo, 12210 Athens, Greece; Email: gfouka@teiath.gr; 3 Technological Educational Institute of Messolonghi, 30200 Messolonghi, Greece; Email: bgna@teimes.gr

**Keywords:** self-rated health, determinants, lifestyle, sleep, religiosity, cross-sectional

## Abstract

Self-rated health (SRH) is a health measure related to future health, mortality, healthcare services utilization and quality of life. Various sociodemographic, health and lifestyle determinants of SRH have been identified in different populations. The aim of this study is to extend SRH literature in the Greek population. This is a cross-sectional study conducted in rural communities between 2001 and 2003. Interviews eliciting basic demographic, health-related and lifestyle information (smoking, physical activity, diet, quality of sleep and religiosity) were conducted. The sample consisted of 1,519 participants, representative of the rural population of Tripoli. Multinomial regression analysis was conducted to identify putative SRH determinants. Among the 1,519 participants, 489 (32.2%), 790 (52%) and 237 (15.6%) rated their health as “very good”, “good” and “poor” respectively. Female gender, older age, lower level of education and impaired health were all associated with worse SRH, accounting for 16.6% of SRH variance. Regular exercise, healthier diet, better sleep quality and better adherence to religious habits were related with better health ratings, after adjusting for sociodemographic and health-related factors. BMI and smoking did not reach significance while exercise and physical activity exhibited significant correlations but not consistently across SRH categories. Our results support previous findings indicating that people following a more proactive lifestyle pattern tend to rate their health better. The role of stress-related neuroendocrinologic mechanisms on SRH and health in general is also discussed.

## 1. Introduction

Self–rated health (SRH) is a health measure based upon a simple question asking individuals to rate their general health in a four or five-point scale. It is considered an inclusive measure of health, meaning that SRH yields information inaccessible by targeted health measurements, something that has increased its popularity in population-based and clinical studies [1]. As such, according to large population-based studies, SRH is a powerful predictor of future health and utilization of health care services [2,3,4]. Moreover, in clinical studies poor SRH has been associated with negative clinical outcomes such as higher mortality and worse patients’ quality of life [1]. As far as general practice is concerned, SRH is proposed as a screening tool for general health assessment, with negative ratings warranting further investigation by practitioners [1]. In support of that, negative health ratings seem to represent pathogenetic biological processes in the body that compromise health status and may herald future health adversities (“interoception hypothesis”) (for a review of SRH see [1]).

Identifying SRH determinants in different populations (e.g., different countries, rural or urban, *etc*.) has several public health benefits. Firstly, cultural and other population characteristics would be more potently addressed by public health policy making, especially as far as the type (rural or urban) of society is concerned. Secondly, deconstructing SRH into its main components enables more targeted interventions and public health policies for a given population. Literature reveals several sociodemographic, disease-related, lifestyle and psychosocial determinants of SRH in different populations. In general, women, individuals with low socioeconomic status and older people tend to rate their health worse, although ambiguity still exists especially for gender [5,6,7]. Moreover, disease-related factors (such as the presence of diseases and physical symptoms, medication usage, hospitalization and utilization of health care services) constitute the principal SRH determinants, with increasing ability to explain SRH variance with ageing [5,8]. Results on lifestyle factors largely vary across studies and populations, but they converge to a common pattern of better health ratings by individuals who adopt a more proactive lifestyle behavior [9,10,11,12].

In the case of Greece, there are limited and parsimonious studies on the determinants of SRH [12,13,14,15,16,17]. The aim of this cross-sectional study is to extend Greek literature by investigating SRH determinants in a rural population. Particular emphasis on lifestyle such as exercise, diet, sleep and religiosity is given. 

## 2. Methods

### 2.1. Sample

This is a cross-sectional study conducted at all the 18 communities of the municipality of Tripoli, Prefecture of Arcadia, Central Peloponnesus, Greece, between January 2001 and December 2003. The study was ethically and methodologically approved by The Research Committee of the Technological and Educational Institute of Athens. As a first step to collect our sample, a letter was sent to local newspapers and official services that hold catalogues of registered citizens. The letter described both study aims and measurement information, assuring candidates for the confidentiality of records. After that, road mapping of the whole region was conducted and home visits were performed by health professionals. No eligibility criteria were used. In case, of absence a second visit was arranged. Each participant gave his/her informed consent before the interviews. The final sample consisted of 1,519 participants out of a population of 4,100 citizens (37.05%), according to 2001 census.

### 2.2. Measurements

#### 2.2.1. Dependent Variable

SRH was evaluated with the following question: “In general, you would describe your health as…”. There were five possible answers: “excellent”, “very good”, “good”, “fair”, “poor”. For the analysis we used three categories: “very good” (excellent/very good), “good” (good) and “poor” (fair/poor). “Poor” SRH served as the reference category in the analyses.

#### 2.2.2. Independent Variables

Sociodemographical variables: gender, age (grouped in three categories: ≤45, 46–65, ≥65 years old), education level (categories: primary ≤6 years, secondary 7–12 and tertiary level >12 years of education).

Health status was assessed with the following open question: “Do you currently suffer from a condition needing regular medical treatment?”. Individuals were asked to list their health problems and two doctors independently classified their answers as valid or not. Classification was the following: healthy (or disease free), one disease needing regular medication and finally co morbidity was defined as the presence of two or more diseases needing regular medication. Each individual had his/her height and weight measured at the site of first encounter. All weighing machines were of the same brand name and model. Body mass index (BMI) was calculated as (Kg/m^2^), according to World Health Organization recommendations, and comprised of three categories for both men and women: normal (<25 kg/m^2^), overweight (25–29.9 kg/m^2^) and obese (≥30 kg/m^2^).

Lifestyle variables included smoking, exercise, diet, sleep and religiosity. *Smoking* was classified as no or yes. *Exercise* was grouped as no or yes regular exercise, with people reporting no planned walking allocated in the no group, while any other planned exercise, including walking, was considered eligible for the yes group. This approach of “planned exercise” was selected in order to avoid the putative bias of daily physical activity related to rural living (e.g., agricultural tasks, *etc*.). *Diet* was evaluated with six questions, each scored as 1 or 0 (Mediterranean diet guidelines were used for scoring purposes): (1) “How would you evaluate the amount of your daily food consumption?” (if high/low = 0, if moderate = 1); (2) “How many times do you eat during the day?” (if ≥3 = 1, if <3 = 0); (3) “How many days per week do you eat red meat?” (if ≤1 = 1, if else = 0); (4) “How many days per week do you eat legumes?” (if ≥1 = 1, if else = 0); (5) “How many days per week do you eat fruits?” (if ≥7 = 1, if else = 0); (6) “How many days per week do you eat vegetables?” (if ≥7 = 1, if else = 0). Total diet score was calculated by summing up answers (range 0–6), with higher scores indicating healthier diet patterns. The 33.3% percentiles were used for categorization into low (score ≤3), moderate (score 4) and high (score 5+) healthy dieters. Similarly, *sleep quality* was evaluated by five questions: (1) “How would you evaluate your sleep?” (if deep = 2, if superficial = 1, if bad = 0); (2) “Is it easy for you to fall asleep?” (if yes = 1, if no = 0); (3) “Do you sleep during midday?” (if always = 2, if sometimes = 1. if never = 0); (4) “Do you feel rest in the morning after awakening?” (if yes = 1, if no = 0); (5) “Are you satisfied with your sleep?” (if yes = 1, if no = 0). Total sleep score was calculated by summing up answers (range 0–7), with higher scores indicating higher sleep quality. The 33.3% percentiles were used for categorization into low (score ≤4), moderate (score 5) and high (score 6+) sleep quality. *Religiosity* was assessed by two simple scored questions: (1) “Do you pray regularly?” (if yes = 1, if no = 0); (2) “How often do you attend church ceremonies?” (if at least once per week = 2, if 3 or less per month = 1, if never = 0). Total religiosity score was calculated by summing up answers (range 0–3), with higher scores indicating higher religiosity. Three religiosity categories were formed: low (score 0 or 1), moderate (score 2) and high (score 3).

### 2.3. Statistical Analyses

Statistical calculations were performed using SPSS for Windows (version 18.0.3) statistical software [18]. Univariate analyses of the independent variables categories with SRH categories was performed using chi-square statistics. Table 1 presents descriptive statistics (absolute and percentage values) of the independent variables across SRH categories, the *p* values of chi-square tests and Kendall's tau b to assist directionality interpretation. Sociodemographic and disease-related variables significant at the p level of <0.1 were first separately and then as a group entered in multinomial regression model with SRH as dependent variable. In general multinomial regression analysis helps to assess the “distance” between very good to poor and good to poor. Statistical significance of a factor in both these comparisons reveals its contribution mainly to negative SRH ratings, whereas significance in very good *vs*. poor only reveals its significance to positive SRH ratings. In other words, factors of the first paradigm could represent already attained or granted situations that are lost, while factors of the second paradigm could represent less granted situations that are gained in the benefit of the individual. Unadjusted and adjusted odds ratios (OR) and 95% confidence intervals (95% CI) are presented in Table 2. Nagelkerke R square represents the amount of SRH variability explained by the sociodemographic and disease-related variables. Finally, using multinomial regression analyses with SRH as dependent variable, each lifestyle variable was adjusted for the effects of the main model (Table 3). The *p* level of significance was set at 0.05 for all analyses.

**Table 1:** Univariate analyses of sociodemographic, disease-related and lifestyle variables with SRH categories (N = 1,519) (chi-square tests).

	Very good	Good	Poor	*p* value (Kendall’s tau b) ^1^		Very good	Good	Poor	*p* value (Kendall’s tau b) ^1^
**Sociodemographic variables**					**Lifestyle variables**				
**Gender**				0.003 * (0.079 *)	**Smoking**				<0.0005 ** (−0.109 **)
Male (%)	231 (35.4)	341 (52.3)	80 (12.3)		No (%)	356 (29.7)	637 (53.2)	204 (17)	
Female (%)	258 (29.9)	449 (52)	157 (18.2)		Yes (%)	133 (41.7)	153 (48)	33 (10.3)	
**Age categories**				<0.0005 ** (0.291 **)	**Regular exercise**				<0.0005 ** (−0.198 **)
≤45 (%)	251 (52.4)	204 (42.6)	24 (5)		No (%)	393 (29)	733 (54)	231 (17)	
46–65 (%)	115 (27.9)	234 (56.8)	63 (15.3)		Yes (%)	96 (60.4)	57 (35.8)	6 (3.8)	
≥66 (%)	123 (19.7)	352 (56.3)	150 (24)		**Healthy Diet categories**				<0.0005 ** (−0.077 **)
**Education categories**				<0.0005 ** (−0.221 **)	Low (%)	231 (27.6)	473 (56.5)	133 (15.9)	
Primary (%)	151 (22.4)	377 (55.9)	147 (21.8)		Moderate (%)	152 (36.7)	203 (49)	59 (14.3)	
Secondary (%)	251 (38.3)	329 (50.2)	75 (11.5)		High (%)	105 (39.8)	114 (43.2)	45 (17)	
Tertiary (%)	84 (51.9)	69 (42.6)	9 (5.6)		**Quality of Sleep categories**				<0.0005 ** (−0.12 **)
**Disease-related variables**					Low (%)	202 (28.6)	344 (48.7)	161 (22.8)	
**Health status**				<0.0005 ** (0.275 **)	Moderate (%)	138 (36.5)	200 (52.9)	40 (10.6)	
Healthy	328 (43.6)	365 (48.5)	59 (7.8)		High (%)	148 (34.7)	244 (57.1)	35 (8.2)	
One disease	114 (26.8)	228 (53.6)	83 (19.5)		**Religiosity categories**				0.016 * (0.039)
Co-morbidity	47 (13.9)	197 (58.1)	95 (28)		Low (%)	99 (37.9)	114 (43.7)	48 (18.4)	
**BMI categories**				0.125 (0.044 ^†^)	Moderate (%)	353 (31.9)	588 (53.1)	166 (15)	
Normal (%)	192 (35.8)	263 (49.1)	81 (15.1)		High (%)	36 (24.7	87 (59.6)	23 (15.8)	
Overweight (%)	187 (31.7)	324 (55)	78 (13.2)						
Obese (%)	101 (29.3)	185 (53.6)	59 (17)						

^1^ Kendall’s tau b coefficient is a statistic used to measure the association between two quantities. In this paradigm positive values signify worse SRH as we vertically advance to independent variables' categories and vice versa for the negative values; ** Level of significance <0.001; * Level of significance <0.05; ^†^ Level of significance <0.1.

**Table 2:** Multinomial regression analyses of sociodemographic and disease-related variables with SRH as dependent variable (N = 1,519).

	Unadjusted Odds Ratios (95% CI)		Adjusted Odds Ratios (95% CI) ^1^	
	Very good *vs*. Poor	Good *vs*. Poor	Very good *vs*. Poor	Good *vs*. Poor
**Gender** (Male *vs*. Female)	1.76 (1.27–2.43) *	1.49 (1.1–2.02) *	1.79 (1.24–2.58) *	1.53 (1.09–2.12) *
**Age categories**				
≤45 *vs*. >65	12.75 (7.88–20.65) **	3.62 (2.28–7.56) **	5.27 (2.9–9.59) **	2.21 (1.25–3.91) *
46–65 *vs*. >65	2.23 (1.51–3.28) **	1.58 (1.15–2.22) *	1.89 (1.25–2.88) *	1.52 (1.06–2.18) *
**Education categories**				
Primary *vs*. Tertiary	0.11 (0.05–0.23) **	0.34 (0.16–0.69) *	0.44 (0.2–0.96) *	0.67 (0.31–1.44)
Secondary *vs*. Tertiary	0.36 (0.17–0.75) *	0.57 (0.27–1.2)	0.5 (0.23–1.06) ^†^	0.69 (0.33–1.46)
**Health status**				
Healthy *vs*. Co morbidity	11.24 (7.19–17.55) **	2.98 (2.06–4.31) **	4.43 (2.66–7.38) **	2.09 (1.36–3.2) **
One disease *vs*. Co morbidity	2.78 (1.77–4.35) **	1.33 (0.93–1.88)	2.07 (1.3–3.3) *	1.19 (0.83–1.7)

^1^ Nagelkerke R square 16.6%, meaning that 16.6% of SRH variance is explained by gender, age, education and disease status. ** Level of significance <0.001; * Level of significance <0.05; ^†^ Level of significance <0.1; Reference categories: Female, 65 years old, tertiary, co morbidity, poor SRH.

**Table 3:** Multinomial regression analysis of adjusted lifestyle factors with SRH as dependent variable (N = 1,519).

	Adjusted Odds Ratios (95%CI) ^1^		Nagelkerke R2 change ^2^
	Very good *vs*. Poor	Good *vs*. Poor	
**Smoking***(No vs. Yes)*	1.37 (0.84–2.24)	1.3 (0.82–2.07)	+0.1%
**Regular exercise***(Yes vs. No)*	4.19 (1.75–10.03) **	1.87 (0.78–4.48)	+1.7%
**Healthy diet categories**			+2.1%
*High vs. Low*	1.89 (1.2–2.97) *	0.78 (0.52–1.18)	
*Moderate vs. Low*	1.64 (1.1–2.46) *	0.96 (0.67–1.38)	
**Quality of Sleep Categories**			+2.0%
*High vs. Low*	2.65 (1.68–4.18) **	2.88 (1.89–4.37) **	
*Moderate vs. Low*	1.69 (1.09–2.63) *	1.8 (1.21–2.7) *	
**Religiosity categories**			+0.9%
*High vs. Low*	2.03 (1.01–4.05) *	2.53 (1.38–4.63) *	
*Moderate vs. Low*	1.93 (1.22–3.03) *	2.03 (1.34–3.08) **	

^1^ Adjusted for gender, age, education and health status; ^2^ Nagelkerke R2 change above the 16.6% of the adjustment model (see Table 2); ** Level of significance <0.001; * Level of significance <0.05; ^†^ Level of significance <0.1; Reference categories: Yes smoking, no regular exercise, low diet, low quality of sleep, religiosity, poor SRH.

## 3. Results

### 3.1. Univariate Analyses of Independent Variables *vs.* the Three SRH Categories

Among the 1,519 participants, 489 (32.2%), 790 (52%) and 237 (15.6%) rated their health as very good, good and poor respectively (missing 0.2%). Table 1 presents distribution of the sociodemographic, disease-related and lifestyle variables across SRH categories with the chi-square tests and Kendall’s tau b coefficients. Missing values were low with the largest found in education categories (3%). All variables, except BMI, were significantly associated with SRH (*p* < 0.05). In particular, women, older people, lower educational level and suffering from one or more diseases reported worse SRH (Table 1). Concerning lifestyle factors, performing planned regular exercise, healthier diet and better quality of sleep were associated with better SRH. Interestingly, smokers and less religious individuals tended to rate their health better than non-smokers and more religious people (Kendall’s tau b −0.109 and 0.039 respectively).

### 3.2. Multinomial Regression Analysis of Sociodemographic and Disease-Related Variables with SRH as Dependent Variable

Table 2 presents unadjusted and adjusted ORs from the multinomial regression analyses for the main adjustment variables that were found significant at the level of <0.1 in the previous univariate analyses. For very good *vs*. poor SRH comparisons all unadjusted variables were found significant (<0.05) with the tendencies described above. However, adjusted ORs decreased considerably for age and disease, although still reached significance. Unadjusted ORs for good *vs*. poor SRH revealed no significant differences between individuals with secondary *vs*. tertiary education and suffering from one disease *vs*. two or more (OR 0.57 95% CI 0.27–1.2 and OR 1.33 95% CI 0.93–1.88, respectively). In the multivariate model, gender, age, education and ill-health status accounted for the 16.6% of the SRH variance.

### 3.3. Multinomial Regression Analyses of Adjusted Lifestyle Variables with SRH as Dependent Variable

Table 3 presents the contribution of each lifestyle variable to SRH, after adjusting for gender, age, education and health status. According to R square increases, lifestyle factor contribution to SRH is the following (in a decreasing order): diet (2.1%), quality of sleep (2%), regular exercise (1.7%), religiosity (0.9%) and smoking (0.1%). Contrary to univariate analyses (Table 1), smoking did not reach significance after adjustment for sociodemographic and disease variables. Healthy diet, either of high or moderate category *vs*. low (OR 1.89 95% CI 1.2–2.97, OR 1.64 95% CI 1.1–2.46, respectively), and regular exercise (OR 4.19 95% CI 1.75–10.03) differed between very good *vs.* poor SRH but not good *vs*. poor. On the other hand, higher quality of sleep (both high and moderate *vs*. low) was associated with better (both very good and good *vs*. poor) SRH (Table 3). Interestingly, after adjustment for main variables, the association of religiosity with SRH was inversed compared to the univariate analysis (Table 1), indicating that more religious participants (both high and moderate *vs*. low) rated their health better (both very good and good *vs*. poor) (Table 3). After performing auxiliary univariate analysis (chi-square) of religiosity with the adjustment variables, we found that women, older people, individuals with lower education and suffering from one or more diseases were significantly (*p* < 0.001) related with greater religiosity.

## 4. Discussion

In this cross-sectional study we investigated the determinants of SRH in a rural population of Greece, giving emphasis on lifestyle factors. Sociodemographic and disease-related variables accounted for 16.6% of SRH variance. In particular, women, older, lower educated and people with impaired health reported worse SRH. According to adjusted ORs, age and health status were more potent determinants of SRH than gender and education. Concerning lifestyle factors, a more proactive lifestyle, as identified by the presence of regular exercise, healthier diet and better quality of sleep, was related with better health ratings. Finally, higher religiosity was associated with better SRH. Considering the “distances” of very good *vs*. poor and good *vs*. poor under the assumption presented in the methods section, female gender, older age, impaired disease status, low sleep quality and low religiosity were more capable to predispose people to fair or poor health ratings (thus considered granted factors that are lost), whereas higher education, less planned exercise and better quality of diet were more capable to predispose people to excellent or very good health ratings (thus considered less granted situations that are gained in the benefit of the individual).

Gender differences on SRH have been confirmed by previous studies [6]. However after controlling for functionality, physical symptoms, health status, emotional distress and locus of control gender differences are eliminated [6,19]. This may indicative of both cultural and gender discrepancies on health evaluation, with different physical, psychosocial factors rendering different populations or genders less or more positive about their health status. Concerning age, previous findings confirm that ageing is linked with worse SRH, although comparative SRH (e.g., comparison with age peers) seems to outweigh global SRH and even improve health ratings in less disabled elders [20]. Low socioeconomic status (SES) (in this study assessed by education level) and impaired health are well established determinants of poor SRH [7]. Although the link between SES and health inequalities is far from doubt, mediators of this relationship still remain elusive. The concept of psychosocial mediators, directly or indirectly linked to stress, seems most promising, since maladaptive stress responses entail a broader range of behavioral and physical changes leading to unhealthy lifestyle patterns and physical “wear and tear”, all jeopardizing health [21].

Exercise and healthy diet are well-known determinants of better SRH [11]. Our study has confirmed such results, even if our measurements were not based on validated physical activity and diet questionnaires. According to our findings, regular exercise and healthier diet are associated with better SRH. More broad associations were found for quality of sleep and religiosity, showing that poor sleep and less adherence to religious habits were associated with poor SRH. Better quality of sleep and high religiousness have been previously reported to positively affect health ratings [22,23]. We have also reported findings of women, older people, individuals with lower education and suffering from one or more diseases being more religious than men, younger, highly educated and healthy people. However, the explained SRH variance by the aforementioned lifestyle variables is minor, implying that other, presumably psychosocial variables such as stress, could account for the residual SRH variance.

There is considerable evidence supporting stress as a putative moderator or mediator of the lifestyle-SRH relationship. Stress is “a state in which homeostasis is actually threatened or perceived to be so” [24]. Excessive stress responses have been linked with maladaptive coping behaviours (such as unhealthy diet, smoking *etc*.) and various non-infectious diseases of modern societies such as diabetes mellitus, metabolic syndrome, cardiovascular diseases, mediated by the emergence of altered psychoendocrinoimmune responses (e.g., elevated cortisol and catecholamines, pro-inflammatory cytokines, *etc*.) [20,24]. Interestingly, stress has been associated with poor SRH and mortality [25,26]. Moreover, poor SRH has been positively associated with chronic stress biomarkers such as urinary epinephrine, salivary cortisol, cortisol to dehydroepiandrosterone sulphate ratio, serum prolactin, high-density lipoprotein (HDL), C-reactive protein, pro-inflammatory immune markers (e.g., TNFa, IL-1, IL-6) and a Th2 shift of immunity [27,28,29,30]. All these stress biomarkers have been also correlated with poor sleep fatigue, morning fatigue and poor psychomotor performance [24,29,31]. Interestingly, good health ratings are favoured by high social support, self-esteem and sense of coherence that buffer the detrimental effects of excessive stress [28].

This study has a number of limitations. Firstly, the cross-sectional design hampers causality inference. Secondly, measurements are self-reports, thus subject to information and recall bias. Seasonal variation in responses during the three-year period of the study may further contribute to information bias. Thirdly, we have used simple questions to address complex factors such as religiosity which is an inclusive variable, integrating not only praying and church attendance but also psychosocial variables pertinent to social networks and social recourses or support. In that way, validity of our measurements may be impaired. For this reason, adding spirituality measurements could be a suitable choice for future studies. On the other hand, it is advantageous for this rural survey that our sample was representative across all the communities studied and included a very large proportion (37.05%) of the total population of Tripoli — A region of the Peloponnese — (census 2001). 

Conclusively, the results of the present and previous studies concur that health status and lifestyle factors are main determinants of SRH in rural populations such as this in Greece. Future studies, should extend research on the role of biological pathogenetic processes and biomarkers on SRH, which could validate the interoception hypothesis mentioned in the introduction. Stress psychology and neuroendocrinology research may pose a new turning point not only of SRH research, but also of public health conceptualization.
